# Hepatitis B Virus Infection, MicroRNAs and Liver Disease

**DOI:** 10.3390/ijms160817746

**Published:** 2015-08-03

**Authors:** Neelakshi Sarkar, Runu Chakravarty

**Affiliations:** ICMR Virus Unit, Kolkata, ID & BG Hospital Campus, Kolkata-700010, India; E-Mail: neelakshisarkar@gmail.com

**Keywords:** HBV, miRNA, chronic, liver cirrhosis, hepatocellular carcinoma

## Abstract

Hepatitis B virus (HBV) attacks the liver and can cause both acute as well as chronic liver diseases which might lead to liver cirrhosis and hepatocellular carcinoma. Regardless of the availability of a vaccine and numerous treatment options, HBV is a major cause of morbidity and mortality across the world. Recently, microRNAs (miRNAs) have emerged as important modulators of gene function. Studies on the role of miRNA in the regulation of hepatitis B virus gene expression have been the focus of modern antiviral research. miRNAs can regulate viral replication and pathogenesis in a number of different ways, which includefacilitation, direct or indirect inhibition, activation of immune response, epigenetic modulation, *etc.* Nevertheless, these mechanisms can appropriately be used with a diagnosticand/or therapeutic approach. The present review is an attempt to classify specific miRNAs that are reported to be associated with various aspects of hepatitis B biology, in order to precisely present the participation of individual miRNAs in multiple aspects relating to HBV.

## 1. Introduction

The hepatitis B virus (HBV) causes acute or persistent liver diseases and, in spite of the availability of an efficient vaccine, it is a major global health problem [1,2]. Discovered in 1966, HBV is a small, enveloped virus with a partially double-stranded DNA genome of the family *Hepadnaviridae* [3]. HBV has affected almost one third of the world’s population and, of them, more than 350 million people have developed chronic HBV infection [4,5,6]. Worldwide HBV infection is a key risk factor for chronic hepatitis, liver cirrhosis, and hepatocellular carcinoma (HCC). The hepatic injuries in HBV infection are thought to be due to immune responses of the host, as HBV is not directly cytopathic. The immune-pathogenesis of hepatitis B depends on an intricate interplay of host factors (such as age, gender, immune status), viral and environmental factors. The molecular determinants that could facilitate the prognosis of HBV infection are yet to be identified in spite of sustained efforts in understanding the molecular mechanisms.

The microRNAs (miRNAs) were discovered in 1993. miRNAsare small non-coding RNAs of 19–23 nucleotides that play major roles in the regulation of gene expression in all eukaryotes by pairing with the 3′-untranslated regions (UTRs) of target transcripts, resulting in inhibition of translation and/or mRNA degradation [7]. To date, over 2000 mature human miRNAs [8] are known and *in silico* prediction estimates that approximately 60% of human mRNA could betargets of miRNA [9]. miRNAs have been reported to be involved in various physiological and pathological functions, including immune response and tumor genesis. A pioneering study by Croce *et al*. [10] revealed the involvement of microRNAs in several types of cancers. Apart from this, miRNAs are important regulators of numerous virus-host interactions. Increasing studies have established that the replication and propagation of various viruses might be influenced by diverse microRNAs. Such viral families include Herpes, polyomavirus, Adenovirus, Ascovirus, papilloma virus, *etc.* [11]. While some miRNAs facilitate viral replication, some others may even serve as potential anti-viral agents. In this respect, HIV (Human Immunodeficiency virus 1), HCV (hepatitis C virus), and HBV (hepatitis B virus) have been vastly studied [12]. Owing to a complicated life cycle and a complex host response, the role of miRNAs in HBV biology is manifold. HBV modulates the expression of cellular miRNAs in order to increase the potential of its own replication and allow evasion from innate immune responses so as to establish viral persistence and propagation in infected hepatocytes [9]. Deregulated expression of miRNAs has been associated with HBV-related pathogensis through the modulation of signaling pathways such as immune responses, apoptosis, proliferation, and migration. Recently it was discovered that extracellular miRNAs are capable of circulating in the serum and these miRNAs are highly stable [13]. On account of their potential to communicate between cells inside the liver, circulating miRNAs are emerging as a growing field in HBV diagnosis as well as therapeutics.

## 2. Biogenesis and Functions of miRNAs

**Figure 1:**
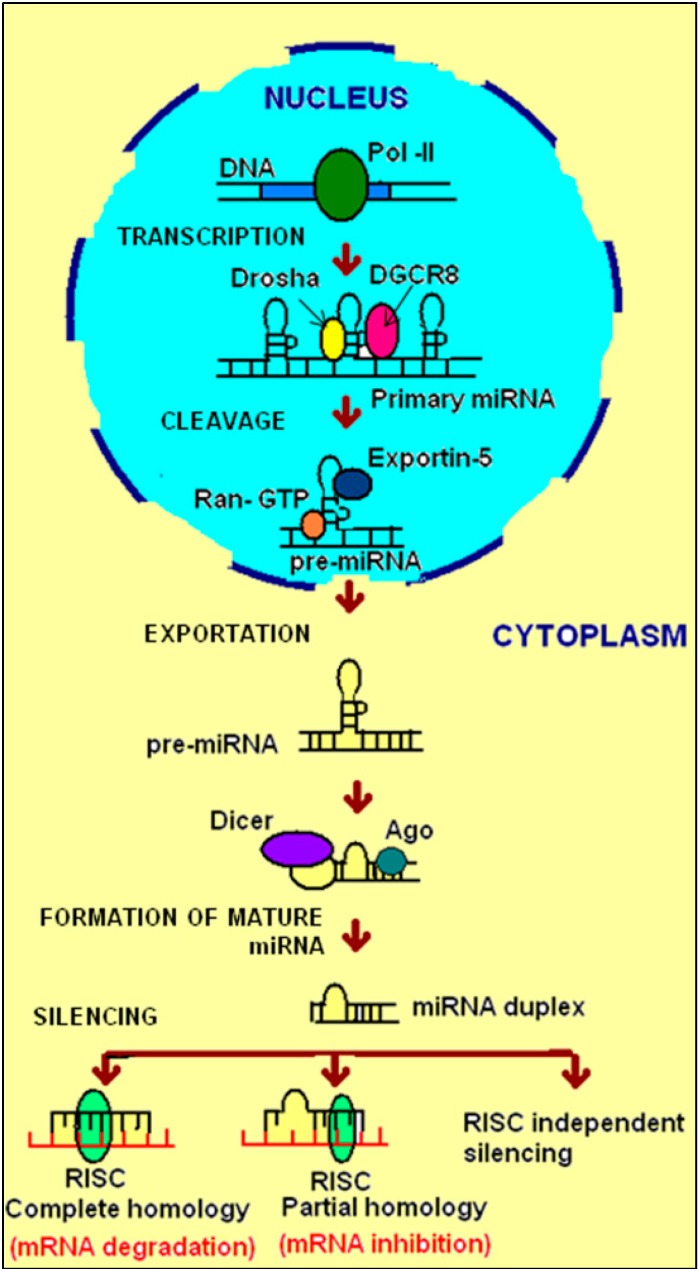
Biogenesis and function of miRNAs. miRNAs are transcribed from specific genes by RNA polymerase II, followed by their cleavage with enzymes Drosha and DGCR8 in the nucleus. The cleaved miRNA is then transported to the cytoplasm by Expotin 5 and Ran GTP where it is further acted upon by the enzyme Dicer to form the mature miRNA duplex. Each strand of the duplex then mediates silencing by either complete base-pairing or incomplete base-pairing with the target mRNA. This reaction is catalyzed by a couple of enzymes which together form the RNA-induced silencing complex (RISC).

The biogenesis of miRNAs is a complex process requiring several steps. Briefly, the first primary miRNAs (pri-miRNAs) transcribed by RNA polymerase II and III in the nucleus are excised subsequently by the ribonuclease Drosha-DGCR8 complex to produce hair-pin precursor miRNAs (pre-miRNAs) in the nucleus. These pre-miRNAs are transported by Ran-GTP and exportin-5 to the cytoplasm, where they are excised by the RNAse III enzyme called Dicer, leading to the production of a miRNA duplex. This duplex in turn splits in order to create the single-stranded mature miRNA that finally forms the RNA-induced silencing complex (RISC). Jointly, RISC and Argonaute/EIF2C (AGO) proteins facilitate the target-mRNA recognition. Identification of target mRNA by miRNA occurs through specific base-pairing interactions between the 5′ end (seed sequence) of miRNA and sites within coding and untranslated regions (UTRs), especially 3′-UTR of mRNAs. At the final point, the mature miRNA either stimulates the repression of translational mechanisms or induces mRNA degradation, depending on the target gene sequence. In other words, if there is a partial complementarity between the miRNA and target mRNA, it will result in the repression of translation, whereas if there is a perfect complementarity, it results in the degradation of the target mRNA [7]. The mRNA degradation, promoted by miRNA, is by induction of de-adenylation or suppression of protein synthesis by repressing the initiation of translation at the cap recognition or inducing ribosomes to drop off prematurely. The mature miRNA can also augment the expression of the target genes, even under circumstances of growth arrest in the cell [9]. Finally, it has been recently reported that miRNA can interact with ribonucleoprotein or directly bind to DNA and establish transcriptional silencing in a RISC-independent manner [14] (Figure 1).

## 3. Viral miRNA and Viral Proteins in Regulation of Host Gene Expression

Although there are a number of viruses that produce specific miRNAs in order to manipulate the host, to date there is no experimental evidence that confirms the synthesis of miRNAs by HBV. However, computational analysis by Jin *et al.* [15] suggested that HBV encodes for only one candidate pre-miRNA, which targets its own mRNA rather than a host mRNA. There are a vast number of studies that recognized that HBV modulates different host miRNAs, but there are only a few reports that established the role of individual viral proteins in the said modulation. Investigations have revealed that, among all viral proteins, hepatitis B X protein (HBx) in particular functions in the regulation of host miRNAs. A microarray executed by Wang *et al.* [16] revealed an up-regulation of seven miRNAs and a down-regulation of 11 host miRNA in HBx-expressing cells. HBx targets miRNAs of the let-7 family (let-7a, let-7b, and let-7c), which in turn regulates the expression of Ras, HMGA2, MYC, and STAT3, resulting in the modulation of host oncogenesis and immune response [16,17,18,19]. HBx protein was also found to up-regulate miR-29a, which in turn enhances cell migration by targeting PTEN in hepatoma cell lines [20]. Additionally, HBx can down-regulate miR-101, resulting in aberrant DNA methylation by targeting DNA methyl transferase3A [21]. Our recent study showed that the expression of tumor suppressor miR-145 and onco-miRs miR-21 and miR-222 are modulated differentially by the HBx protein in malignant hepatocytes [22]. Furthermore, it has been found that the up-regulation of miRNA-146a by HBx protein contributes to hepatitis development through down-regulation of complement factor H [23]. In a study, Wang *et al.* [24] showed that, unexpectedly, HBx transcript can directly mediate the repression of miR-15a/miR-16-1 without the essential requirement of HBx protein, thus indicating the vital role of the HBx gene in host miRNA regulation.

## 4. Role of Host miRNAs in Viral Infection

The host-viral interplay has long been exciting the field of research across the world. Conflicting efforts of the virus (to manipulate the host for its successful propagation) as well as the host (to inhibit the virus) culminate in complex interactions resulting in either elimination of the virus or its persistence. miRNAs play a vital role in such interactions. Several studies to date have revealed that there is a large number of host miRNAs that can regulate HBV replication in numerous ways. Mainly, such miRNAs inhibit viral replication. Among them, certain miRNAs directly target viral RNA to block its replication; others target specific host genes which in turn hinders viral propagation. Some miRNAs are even capable of activating specific immune responses in order to restrain the virus.

### 4.1. MiRNAs that Facilitate Viral Replication

It is noteworthy that there is no strong evidence that shows host miRNAs directly facilitate viral replication. However, there is fleeting evidence that some miRNAs can enhance viral replication upon their up-regulation. For instance, Dai *et al.* [25] reported that miR-15b promotes HBV replication by aiding HBV enhancer I activity through human hepatocyte nuclear factor 1α (HNF-1α), but HBx expression represses the expression of miR-15b. Another study by Jin *et al.* [26] showed that down-regulation of miR-501 significantly inhibited HBV replication. They demonstrated that HBx-interacting protein (HBXIP), a HBV replication inhibitor, is a potential target of miR-501, the repression of which activates HBV replication. Again, Zhang *et al.* [27] reported that miR-1 enhanced HBV core promoter activity by promoting the expression of farnesoid X receptor α. There is also an independent study establishing that miRs-372/373 stimulated the production of HBV proteins and HBV core-associated DNA in HepG2 cells by targeting nuclear factor I/B [28]. There are also reports which disclose that even miR-146a and miR-548 promote HBV replication by repressing specific host immune factors [29,30].

### 4.2. MiRNAs that Directly Inhibit Viral Replication

These are miRNAs with target sequences in the viral genome and therefore they can directly target and inhibit HBV without any intermediate factors. In a study, Wu *et al.* [31] applied computational analysis to determine miRNAs with putative target sites in the HBV genome. He screened out let-7, miR-196b, miR-433, miR-511 (targets polymerase and surface genes), miR-205 (target HBX), and miR-345 (targets pre-core genes). Though he found that their target regions were highly conserved across various clades of HBV, this analysis requires experimental validation. However, there are also a number of other miRNAs that exhibit direct interactions with viral components and such interactions have been validated experimentally. These include miR122 (targets the coding region of the mRNA for viral polymerase and the 3′-UTR region for the core protein of the HBV genome) [32], miR199a-3p (targets the HBsAg coding region) [33], miR210 (targets the HBV pre-S1 region) [27], miR125a-5p (interacts with the HBV surface antigen and inhibits its expression) [34], miR15a (targets HBx transcript) [24], miR20a, miR-92a-1, miR-16-1 (HBV transcripts) [35], and miR205 (targets X gene) [36].

### 4.3. MiRNAs that Indirectly Inhibit Viral Replication

These are comprised of miRNAs that do not have a direct binding site in the HBV genome but can indirectly regulate viral replication/transcription by targeting other host factors associated with the replication of the virus. In this respect, miR122 has been extensively studied. It is a liver-specific miRNA that is involved in a complicated signaling network that affects HBV infectivity. While some studies acknowledge that miR122 enhances viral replication, others suggest that it inhibits viral replication. For instance, miR122 promotes viral replication by targeted inhibition of heme oxygenase-1 [37]. On the other hand, it targets CyclinG1 and NDRG3 (a member of the N-myc downstream-regulated gene) to inhibit viral replication [38]. Wang *et al.* [39] reported that miR-29c functions in the suppression of HBV replication by targeting TNFAIP3, a chief regulator of inflammation and immunity. Additionally, ectopic expression of miR-125b inhibits HBV DNA intermediates and the secretion of HBsAg and HBeAg by targeted repression of SCNN1Α (sodium channel, non-voltage-gated 1 alpha) [40]. Similarly, miR-141 and miR155 can repress HBV replication effectively through direct targeting of PPARα (peroxisome proliferator activated receptor alpha) [41] and C/EBP-β (CAAT Enhancer binding protein β) [42], respectively. A recent report describe that MiRNA-130a represses hepatitis B virus replication via targeting PGC1α and PPAR which are HBV transcription enhancers [43].

## 5. MiRNAs Associated with Liver Disease

HBV is a non-cytopathic virus with a complicated life cycle. Emerging research has embarked upon the specialized roles of specific miRNAs that can be associated with different phases of HBV infection. However, these reports are sometimes contradictory primarily on account of the specimens used for the study (whether Peripheral blood mononuclear cells or liver biopsy or serum). It is worth mentioning that liver diseases (chronic liver disease, liver cirrhosis, and hepatocellular carcinoma) might also occur on account of multiple other infections (HCV, HDV, HAV, HEV) and environmental factors (e.g., alcoholic liver disease, *etc.*) and there are miRNAs that specifically mark the clinical stage of liver disease irrespective of the etiology. However, among them, the miRNAs that are particularly modulated in HBV-related liver diseases are discussed below.

### 5.1. Chronic Hepatitis

Increasing studies have revealed an association of miR122, a liver-specific miRNA, with chronic liver diseases. Reports indicated that miR122 is up-regulated significantly in blood samples of HBeAg-positive chronic hepatitis patients [13]. Another report showed that the levels of miR372/373 rise with increasing HBV DNA titer and thus can serve as a marker of chronic hepatitis [28]. An interesting study by Chen *et al.* [44] revealed that let-7c, miR23b, miR122, and miR150 could serve for the diagnosis of occult HBV infection.

### 5.2. Liver Cirrhosis

Of all miRNAs that have been associated with cirrhosis of the liver, miR29 has been particularly well-acknowledged [45,46,47,48]. Members belonging to the family of miR29 have been reported to be negatively associated with advancing cirrhosis/fibrosis as well as with the degree of necro-inflammation [46]. Cirrhosis primarily occurs due to collagen deposits secreted by hepatic stellate cells (HSCs) in the extracellular matrix of hepatocytes. Reports indicated that miR29 suppresses cirrhosis/fibrosis upon its up-regulation by repressing collagen-secreting genes in HSCs through TGF-β and NF-κB pathways [45,47,48]. Other miRNAs known to be down-regulated during cirrhosis/fibrosis include miR133a and miR199 [49,50]. However, miR181b, miR214-5p, miR221, and miR222 are up-regulated during cirrhosis and can thus function as markers [51,52].

### 5.3. Hepatocellular Carcinoma

The reports pertaining to HBV-related HCC are manifold. HBV, particularly the HBx protein, has been reported to modulate the expression of different cellular miRNAs and facilitate hepatocarcinogenesis. Connolly *et al.* [53] identified that the miR-17-92 cluster (mir-17-5p, miR-18a, miR-19a, miR-19b, miR-20a, and miR-92a-1) was elevated in HBV-associated HCC. He found that this up-regulation, primarily facilitated by the HBX protein, is responsible for progression to HCC. In the same study he also found miR21 to be associated with HBV-related HCC [53]. On the other hand, Laderio emphasized upon miR-96,which he reported to be significantly over-expressed in HBV-related HCC as compared to non HBV-related HCC [54]. Li *et al.* [55] performed Solexa sequencing followed by validation with quantitative RT-PCR assay to examine miRNA associated with HBV- and HCV-related HCC. He found that compared to the control, 13 miRNAs were differentially expressed in HBV-associated HCC cases, which accurately discriminated HBV sera samples from controls and HCV cases. Among them, he identified miR-375 and miR-92a to be particularly HBV-specific. Gao *et al.* [56] working with small HCCs and dysplastic nodules (DNs) in the early stages of HBV-associated HCC, found that miR-145 and miR-199b were down-regulated while miR-244 was up-regulated in premalignant DNs, which persisted all the way through the development of HCC. In addition to this, Wang *et al.* [57] described miR155 to be up-regulated in HBV-related HCC. Another independent report demonstrated that HBx protein hypermethylates CpG islands on the miR-122 promoter and thereby disrupts the binding of peroxisome proliferator activated receptor gamma (PPARγ) and retinoid X receptor alpha (RXRα), resulting in deregulated miR122 expression during HCC [58]. HBx also regulates the expression of miR-205 and miR-132 through induction of promoter hypermethylation [36,59]. HBx also facilitates hepatocarcinogenesis through suppression of the master tumor suppressor p53, resulting in decreased synthesis of miR-34, miR-23a, miR-148a, miR-192, miR-125b, and miR-200 [35,60,61,62,63,64]. These miRNAs generally inhibit carcinogenesis, therefore, if de-regulated, they can facilitate HCC progression [35,60,61,62,63,64]. Studies also established that miR-602 progressed to increase significantly from the stage of HBV infection and to reach its peak in HCC when compared with healthy liver [65]. RASSF1A protein was identified as a target of miR-602 that could facilitate apoptosis and inhibit cell proliferation.

## 6. miRNAs in Epigenetic Regulation of HBV

Epigenetic modifications, such as promoter methylation or histone acetylation, have been established to affect miRNA expression [66].Conversely, many miRNAs can themselves control the expression of key epigenetic components in a tightly regulated feedback mechanism. miRNAs related to epigenetic modulation were defined as “epi-miRNAs”. For instance, miR-1 has been demonstrated to target and inhibit HDAC4, a member of histonedeacetylase [27]. Since HDAC suppresses HBV replication, up-regulation of miR-1 promotes the replication of HBV. Additionally, miR-29c exerts a suppressive action on tumors and is down-regulated in several types of cancer, including HBV-associated hepatocellular carcinoma. MiR-29 directly targets DNA methyltransferase 3A and 3B, two vital enzymes involved in DNA methylation and the silencing of tumor suppressor genes [67]. Furthermore, miR-152 could target DNA methyltransferase-1 (DNMT-1) and reduce its expression [68], which in turn affects HBV gene expression and replication by promoting viral DNA methylation.

## 7. miRNA Polymorphism and HBV Infection

Since miRNAs are intricate regulators of global gene function, it is not surprising that single-nucleotide polymorphisms (SNPs) in miRNA encoding genes can be associated with the susceptibility to HBV persistence and, consequently, HCC development. Emerging studies have reported variable SNPs in different miRNA encoding genes. However, among them, several reports are contradictory owing to the differences in ethnic background of the study isolates. In a case-control study from Korea, Cheong *et al.* [69] found that the T allele on the miR-604 gene served as a protective factor for the occurrence of HCC in patients with HBV-related chronic liver disease. In another independent study they found the T allele in the miR-219-1 promoter to be strongly associated with HBV clearance [70]. On the other hand, Kou *et al.* [71] found that the miR-196a2C>T polymorphism imparts protection in HBV-related HCC. Yu *et al*. [72] found an association of microRNA-323b polymorphism with the persistence of hepatitis B virus. They established, through *in vitro* experimentation, that this polymorphism facilitated the enhancement of viral replication. In yet another study, Xiang *et al.* [73] indicated that the miR-499 polymorphism was associated with the susceptibility to HBV-related HCC in the Chinese population. A meta-analysis by Xu *et al.* [74] revealed three common functional SNPs in miRNA-encoding genes, namely miR-146a G → C (rs2910164), miR-196a-2 C →T (rs11614913), and miR-499 T → C (rs3746444). They demonstrated that SNPs contained in the genes encoding miR-146a and miR-196a-2 may play a major role in genetic susceptibility to HCC, whereas the miR-146a C variant was associated with a decrease in HCC risk, especially among Asian and male populations; the miR-196a-2 T variant, however, was associated with susceptibility to HCC among Caucasian populations. The study by Su *et al*. [75] also supports that miR-146a polymorphism plays a possible role in genetic susceptibility to HBV-related HCC among Asians.

## 8. miRNAs and Host Immune Response

Studies relating to the role of miRNAs in host immune response against HBV are limited. To date, only a few miRNAs have been experimentally established to be linked to immune molecules associated with HBV. Su *et al.* [76] suggested that miR155 targets SOCS1 and thus enhances innate antiviral responses against HBV through the JAK-STAT pathway. Our lab reported that the expression of miR155 positively correlates with Toll-like receptor 7 expression during hepatitis B infection [42]. Moreover, miR-34a has been reported to target CCL22 and suppress HBV infection by inhibiting regulatory T cells [60]. On the other hand, miR-146a and miR-548 targets STAT1 and IFN-λ1, respectively, to promote HBV infection [29,30].

Interestingly Jiang *et al.* [76] conducted a microarray in HepG2 *vs.* HepG2.2.15 cells and tried to find out the link between significantly up-regulated miRNAs and Toll-like receptor (TLR) signaling pathways. They screened out miR-200b-3p, miR-148a-3p, miR-145-5p, miR-146b-5p, miR-200c-3p, miR-455-3p, and miR-455-5p, which were highly up-regulated in HepG2.2.15 cells as well as associated with Toll-like receptor signaling pathways [77]. They affirmed that miRNAs and their regulation play a significant role in HBV infection, and HBV may control the TLR signaling pathway through the modulation of miRNAs. Though this is a significant step in understanding the role of TLRs and miRNA in HBV infection, this requires experimental validation.

## 9. Circulating miRNAs as Biomarkers in HBV Infection

Since HBV is a liver disease that can be classified into several clinical categories based on the disease severity, more and more studies focus on the development of biomarkers that could detect the clinical stage of infection. It has been previously established that miRNAs can leak from cells following injury or can be secreted from the cells into circulation [78]. The fact that these circulating miRNAs can stably reside in the serum/plasma of subjects further incited researchers to use them as non-invasive biomarkers. In a recent study, Zhou employed a three-phase validation of an initial microarray result to determine the miRNAs that could be used as biomarkers for liver disease. His findings identified seven miRNAs (miR-122, miR-192, miR-21, miR-223, miR-26a, miR-27a, and miR-801) that were differentially expressed between HCC and control groups [79]. Hayes *et al.* [80] reported that miR-122, miR-22, and miR-99a were up-regulated by 1.5-fold in the serum of HBV-infected patients and thus could be used as disease-specific biomarkers. However, among them, most studies emphasized miR-122, as its levels presented a disease severity-dependent change in patients and, therefore, it is potentially useful as a blood marker for liver injury, including HBV-associated injury [81,82,83]. Reports indicated that miR-122 levels increased and positively correlated with serum ALT (Alanine amino transferase) levels in acute liver disease. Additionally, its levels in the sera were also up-regulated in chronic hepatitis as well as in HCC patients and correlated positively with HBsAg levels. Studies also established that miR122 could serve as a marker of HCC in general, but it could not be used to discriminate HBV-associated and non-associated HCC. It is noteworthy that, though miR122 is decreased upon HBV infection in liver cells, their levels are increased in sera possibly because miR-122 from damaged hepatocytes might accumulate in the blood at high levels [78]. MiR-21 was also adequately studied to explore its possible role as a biomarker. Reports established that miR21 could serve as a biomarker for HCC, as it could differentiate HCC from healthy controls with high sensitivity and specificity [84,85]. Additionally, Li *et al.* [55] demonstrated that miR25, miR375, and let-7f are also important markers of HCC. In another independent report, Li *et al.* [86] suggested the potential role of miR221 as a biomarker as elevated levels of miR-221 were associated with tumor size, tumor stage, cirrhosis, and lower survival rate. It has also been observed that miR223 levels in tissues are depleted during HCC, but its concentration in serum rises, suggesting its use as a serum biomarker of HCC [83]. Other miRNAs that have been studied to evaluate their potential role as biomarkers in liver diseases include miR-16, miR-129-2, and miR-17-5p [87].

## 10. miRNAs in HBV Therapeutics

A number of host miRNAs that may be capable of combating HBV have been reported, but no clinical trial has been conducted on them. The most important concern, as of now, in this respect, is the delivery of the miRNAs, as delivery by an inappropriate procedure would be highly cyto-toxic [88]. In this respect miR122, miR30, and miR31 have been studied. The designing of “pri-miR-like” sequences for the delivery of miR122 and miR31 has been successful in achieving >80% knockdown of viral replication [89]. On the other hand, usage of the endogenous substrate of the miRNA30 processing pathway has potential [90,91]. Another concern in this respect is the management of therapy based on the clinical stage of HBV infection. For instance, miR-29b mimics prevent liver fibrosis [92] while miR-99a mimics significantly inhibited tumor growth during HCC [93]. Further research with appropriate analysis of toxicity in the *in vivo* system, as well as a suitable delivery system, might serve to facilitate future treatment prospects against HBV in the long-term.

## 11. Concluding Remarks

**Figure 2:**
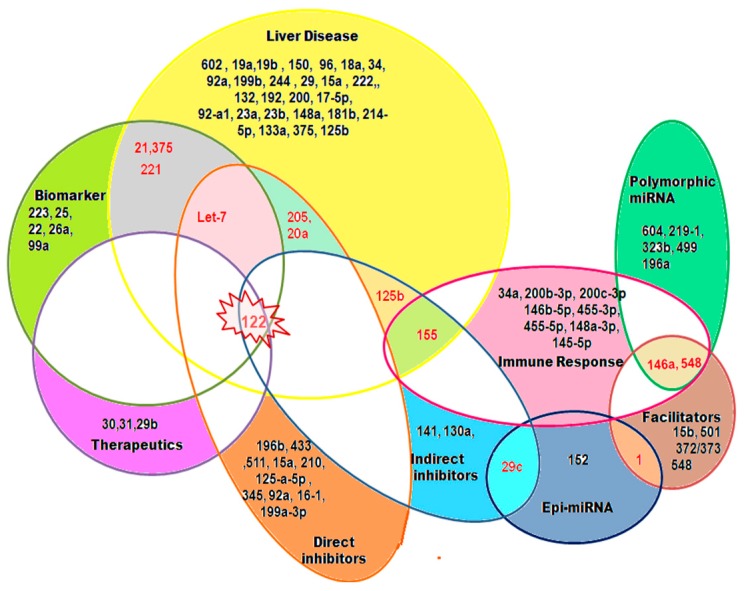
An overview of specific miRNAs associated with different aspects of hepatitis B virus biology. miRNAs are designated by numbers in the Venn diagram. Those marked in red color indicate miRNAs that are associated with multiple aspects of HBV biology as depicted, while the ones marked in black indicate those miRNAs that are associated with a single aspect.

The limitations of using nucleoside analogues as a treatment strategy and the unavailability of an appropriate biomarker indicating the stage of HBV-induced liver disease have long concerned researchers across the world. The discovery of miRNAs as an integral modulator of gene function motivated them to utilize them with an antiviral approach. Since then, large-scale studies have revealed that many host miRNAs are modulated by the virus in order to accomplish its persistence, while other miRNAs are modulated by the host in order to achieve viral clearance. One interesting miRNA in this respect is miR-155, as it is involved in the immune responses against HBV and can thus boost such responses in order to mediate viral repression. Additionally, there is evidence which shows that it can trigger apoptosis in cells [94]. However, some contradictory reports establish an involvement of miR155 in oncogenesis [95], and this is a matter of concern that therefore requires verification. Of the several miRNAs that have been studied in the context of HBV infection, miR122 seems to have a promising role in future diagnosis/therapeutics. This miRNA is involved with several aspects of HBV biology. On one hand, it can directly as well as indirectly hinder HBV replication. On the other hand, its up-regulation in the serum of HCC patients can serve as an effective biomarker. Additionally, it is a tumor suppressor whose ectopic expression can efficiently suppress HBV-associated oncogenesis. Consequently, miR-122 mimics might be developed as a future therapeutic tool. Interestingly, miR-122 promotes hepatitis C virus (HCV) infection and, thus, treatment of HCV requires antagomirs against miR122 (instead of mimics), which have already been tested in clinical trials [96]. Moreover, development of circulating miRNA as biomarkers will possibly help in developing non-invasive strategies for the detection of HBV-associated disease progression. Therefore, in conclusion, the results of several interesting studies up to now and in the future, relating to miRNAs, might help us to conquer the largely unknown and still mysterious menace of chronic HBV infection (Figure 2).
